# Effect of ginseng extract on the TGF-β1 signaling pathway in CCl_4_-induced liver fibrosis in rats

**DOI:** 10.1186/s12906-016-1507-0

**Published:** 2017-01-13

**Authors:** Mohamed M. Hafez, Sherifa S. Hamed, Manal F. El-Khadragy, Zeinab K. Hassan, Salim S. Al Rejaie, Mohamed M. Sayed-Ahmed, Naif O. Al-Harbi, Khalid A. Al-Hosaini, Mohamed M. Al-Harbi, Ali R. Alhoshani, Othman A. Al-Shabanah, Shakir Dekhal Alsharari

**Affiliations:** 1 Department of Pharmacology and Toxicology, College of Pharmacy, King Saud University, P.O. Box 2457, Riyadh, 11451 Kingdom of Saudi Arabia; 2 Department of Zoology, College of Science, King Saud University, Riyadh, Kingdom of Saudi Arabia; 3 Department of Zoology, College of Science, Alexandria University, Alexandria, Egypt; 4 College of science, Zoology and Entomology department, Helwan University, Cairo, Egypt; 5 National Cancer Institute, Virology and Immunology Unit, Cancer Biology Department, Cairo University, Cairo, Egypt

**Keywords:** Ginseng extract, Carbon tetrachloride, Gene expression, Real time PCR

## Abstract

**Background:**

Liver diseases are major global health problems. Ginseng extract has antioxidant, immune-modulatory and anti-inflammatory activities. This study investigated the effect of ginseng extract on carbon tetrachloride (CCl4)-induced liver fibrosis in rats.

**Methods:**

Male Wistar rats were divided into four groups: control group, ginseng group, CCl_4_ group and CCl_4_ + ginseng group. Liver injury was induced by the intraperitoneal (I.P) injection of 3 ml/kg CCl_4_ (30% in olive oil) weekly for 8 weeks. The control group was I.P injected with olive oil. The expression of genes encoding transforming growth factor beta (*TGF-β*), type I TGF-β receptor (*TβR-1*), type II TGF-β receptor (*TβR-II*), mothers against decapentaplegic homolog 2 (*Smad2*), *Smad3*, *Smad4*, matrix metalloproteinase 2 (*MMP2*), *MMP9*, tissue inhibitor matrix metalloproteinase-1 (*TIMP-1*), Collagen 1a2 (*Col1a2*), Collagen 3a1 (*Col3a1*), interleukin-8 (*IL-8*) and interleukin -10 (*IL-10)* were measured by real-time PCR.

**Results:**

Treatment with ginseng extract decreased hepatic fat deposition and lowered hepatic reticular fiber accumulation compared with the CCl_4_ group. The CCl_4_ group showed a significant increase in hepatotoxicity biomarkers and up-regulation of the expression of genes encoding *TGF-β, TβR-I, TβR-II, MMP2, MMP9, Smad-2,-3, -4, and IL-8* compared with the control group. However, CCl_4_ administration resulted in the significant down-regulation of IL-10 mRNA expression compared with the control group. Interestingly, ginseng extract supplementation completely reversed the biochemical markers of hepatotoxicity and the gene expression alterations induced by CCl_4_.

**Conclusion:**

**g**inseng extract had an anti‐fibrosis effect via the regulation of the TGF‐β1/Smad signaling pathway in the CCl_4_‐induced liver fibrosis model. The major target was the inhibition of the expression of *TGF‐β1, Smad2,* and *Smad3*.

## Background

The liver plays a key role in various pathological disorders such as fatty liver, hepatic virus infection, chemical hepatotoxins, and toxicity cases [1, 2]. Several hepatotoxicants including polycyclic aromatic hydrocarbons, nitrosamines, and carbon tetrachloride (CCl_4_) are transformed into intermediate reactive oxygen species that have hepatotoxic effects in humans and experimental animal models [3]. For models of human disease, the rat offers many advantages over mice and other organisms, including the size of its body and substructures in organs, as well as the ability to measure drug effects at specific anatomical areas [1].

Chronic liver diseases are worldwide health problems causing approximately 800,000 deaths per year [2, 3]. Of these, liver fibrosis is caused by inflammation and the excessive accumulation of the extracellular matrix. Subsequently, cirrhosis occurs and can cause hepatocellular carcinoma [4]. Although advanced fibrosis is reversible depending on the degree of fibrosis, end-stage cirrhosis is irreversible [5].

The inhibition of hepatic inflammation and fibrosis is crucial in preventing cirrhosis and HCC. However, the currently used methods for fibrosis evaluation are invasive such as liver biopsy or non-invasive including serum and genetic tests, and imaging techniques [6].

Carbon tetrachloride (CCl_4_) is a hepatotoxin that targets both the liver and kidneys. CCl_4_ is activated in the liver to highly reactive trichloromethyl radicals that initiate the free radical-mediated lipid peroxidation of membrane phospholipids, causing functional and morphological changes in the cell membrane, which stimulate hepatotoxicity, fibrosis [7], cirrhosis and HCC in animal species [8]. Reactive oxygen species results in oxidative stress that plays a significant role in liver fibrogenesis [9]. Tri-chloromethyl radicals produced from CCl_4_ metabolism initiate reactions that cause liver steatosis, fibrosis or cirrhosis [9] via the activation of cytokines such as interleukin (IL)-1, tumor necrosis factor (TNF)-α, and transforming growth factor (TGF)-β expression, and the inhibition of nitric oxide (NO) formation [10–12].

Activated TGF-β stimulated the over expression of many extracellular matrix (ECM) proteins and suppressed their degradation by matrix metalloproteinases (MMP) via the upregulation of tissue inhibitor of metalloproteinases (TIMP) [13]. Binding of TGF-β to TGF-β receptor–II (TβR-II) triggers signals mediated by the phosphorylation of receptor associated Smads (Smad2 and Smad3; R-Smads), which then form a complex with Smad4 that enters the nucleus and binds to the TGF-β promoter to regulate its expression [14].

*Panax ginseng* root is used in oriental medicine, diets or as a dietary supplement. Ginseng has a variety of beneficial biological properties including anti-diabetic, anti-carcinogenic, anti-inflammatory, and neuro-protective effects [15]. The pharmacological properties of ginseng are mainly attributed to ginsenosides, which are phenolic acids, flavonoids, and triterpenoid saponins [16]. Ginsenosides are bioactive compounds such as Rb_1_, Rb_2_, Rg_1_, Rd, and Re [10, 17] that have antioxidant and anti-inflammatory effects [18] via different mechanisms and pathways in vitro, in vivo, and in clinical models [19].

Many studies have shown that ginseng attenuates liver injury by inhibiting lipid peroxidation, as well as TNF-α, NO, prostaglandin E2 (PGE2), intercellular adhesion molecule (ICAM)-1 and nuclear factor-κB (NF-κB) activation [14, 20–22]. However, the pharmacological effects of ginseng/ginsenosides on liver disorders, especially liver fibrosis, are not clear. Therefore, the aim of this study was to investigate the effect of ginseng extract on CCl_4_-induced liver inflammation and fibrosis in rats.

## Methods

The present study was performed and described according to the Animal Research: Reporting In Vivo Experiments (ARRIVE) statement [23].

### Chemicals

CCl_4_ and ginseng (*Panax ginseng*) were purchased from Sigma Chemicals (Sigma-Aldrich, St. Louis, MO, USA). Carbon tetrachloride was dissolved in olive oil, an emulsifying agent that allows CCl4 to dissolve sufficiently to induce liver damage. In addition, olive oil has no toxicity or other biological or pharmacological activity with regard to hepatotoxicity. Ginseng was dissolved in water and administrated by oral gavage. The SYBR® Green PCR Master Mix kit was purchased from Applied Biosystems (Life Technologies, Grand Island, NY, USA). Primers used in this study were designed using Primer Express 3.0 software (Applied Biosystem, Life Technologies, Grand Island, NY, USA) and synthesized by Metabion International AG (Planegg, Germany). Aspartate aminotransferase (AST) and alanine aminotransferase (ALT) measurement kits were purchased from Human (Human, Wiesbaden, Germany).

### Study animals

Six-week-old male Wistar rats with a mean body weight of 180–200 g were obtained from the Animal Care Center, College of Pharmacy, King Saud University, Riyadh, Saudi Arabia. The animals were kept under standard conditions of temperature (22 ± 1 °C), humidity (50–55%), and a 12 h light/dark cycle, with free access to standard laboratory feed and water according to the study protocol. All methods were conducted in accordance with the Guide for Care and Use of Laboratory Animals, Institute for Laboratory Animal Research, National Institute of Health (NIH publication No. 80–23; 1996). The study was approved by the Experimental Animal Care Center Review Board (number E.A.C.P -6140/2016), King Saud University Riyadh, Saudi Arabia.

### Experimental design

The experimental design was based on a previous protocol [24, 25]. Forty adult male Wistar rats were randomly divided into four groups of 10 animals each as follows.

1. Group I: the control group received 3 mL/kg olive oil twice a week for 8 weeks.
2. Group II: the ginseng group received 400 mg/kg/day ginseng dissolved in water administrated by oral gavage for 8 weeks.
3. Group III: the CCl_4_ group received 3 ml/kg CCl_4_ intraperitoneal (30% in olive oil) twice a week for 8 weeks and normal drinking water.
4. Group IV: the CCl_4_-ginseng group received 3 ml/kg CCl_4_ (30% in olive oil) twice a week for 8 weeks and 400 mg/kg/day ginseng dissolved in water and administrated by oral gavage one week before the first dose of CCl_4_ and continuously to the end of the protocol.

At least 24 h after the last treatment, blood samples were collected via cardiac puncture under anesthesia and centrifuged to separate the sera. Then the animals were euthanized by decapitation. The sera were separated and kept at −80 °C until used. The liver was immediately removed and divided into sections that were either washed with ice-cold saline solution, snap frozen in liquid nitrogen and stored until used for gene expression analysis, or cut into small pieces of 0.5–2.0 cm thick for histopathological study.

### Histopathological study

Samples of livers from control and experimental groups were fixed in 10% neutral buffered formalin. The standard method of dehydration, clearing in xylene, and paraffin embedding was used. Sections of 5-μm thickness were cut by a rotary microtome and stained with Masson’s Trichrome (Bancroft and Gamble 2008). Sections were examined by light microscopy.

### Liver biological activities

Blood from the heart was collected into coagulant tubes, left to coagulate, and then centrifuged at 3000 × g for 15 min at 4 °C. Serum ALT and AST activities were measured spectrophotometrically using a commercial kit (Human, Wiesbaden, Germany).

Serum and hepatic triglycerides (TG), total cholesterol (TC), low-density lipoprotein (LDL), and high-density lipoprotein (HDL) concentrations were measured using commercial enzymatic kits (Randox® TR213 for triglycerides, Randox® CH201 for total cholesterol, Randox Laboratories Ltd., London, UK).

Liver samples were homogenized in a chloroform/methanol (2:1) solution to a final dilution 20 times the volume of tissues for at least 3 min and extracted with a chloroform/methanol/water (3:48:47) solution [26].

### Measurement of gene expression by real-time PCR

Total RNA was extracted from liver tissues using a Total RNA Miniprep Kit (Axygen, Bioscience, Central Avenue, Union, USA) according to the manufacturer’s protocol. The RNA concentrations and purity were measured by NanoDrop (NanoDrop 8000, Thermo Scientific, USA). The extracted RNA had a 260/280 ratio of 1.9–2.1. cDNA was synthesized from 1 μg RNA using a high capacity cDNA reverse transcription kit as described in the manufacturer’s protocol (Applied Biosystems, Life Technologies, Grand Island, NY, USA).

Real-time quantitative PCR (SYBR® Green PCR Master Mix kit, Applied Biosystems, Life Technologies, Grand Island, NY, USA) was used to detect the expression levels of transforming growth factor beta *(TGF-β),* type I TGF-β receptor *(TβR-1),* type II TGF-β receptor *(TβR-II),* mothers against decapentaplegic homolog 2 *(Smad2), Smad3, Smad4,* matrix metalloproteinase2 *(MMP2), MMP9,* tissue inhibitor matrix metalloproteinase-1 *(TIMP-1),* Collagen 1a2 *(Col1a2), Collagen 3a1 (Col3a1),* IL-8 *(IL-8)* and IL-10 *(IL-10)* genes in liver tissues. *GAPDH* was used as an internal control. The reaction was performed on ABI 7500 Detection System (Applied Biosystems, Life Technologies, Grand Island, NY, USA). The program was set to run for one cycle at 95 °C for 2 min, followed by 40 cycles at 95 °C for 15 s and 60 °C for 1 min. The specificity of amplification was confirmed by melting curve analysis. The primer sequences used in this study are shown in Table 1. The gene expression results were analyzed using the 2^-ΔΔCT^ method [27]. Data were expressed as the mean fold change ± standard error for three independent amplifications.

**Table 1:** Primers used in this study

Gene name	Forward primer	Reverse primer
*TGF-β*	5’-TAGGCTGACAGCTTTGCGAA-3’	5’-GAACAACCGGCCTCCAAAAC-3’
*TβFI*	5’-GAAATCGCTCGACGCTGTTC-3’	5’-TTCGCAAAGCTGTCAGCCTA-3’
*TβFII*	5’-CGTGTGGAGGAAGAACGACA-3’	5’-CGTGGGAGAAGTGGCATCTT-3’
*Col1a2*	5’-GCCAAGAATGCATACAGCCG-3’	5’-GACACCCCTTCTGCGTTGTA-3’
*Col3a1*	5’-CCCAGGGATTCCAGGACCTA-3’	5’-ACTCCTCTAGGTCCTGCAGG-3’
*IL-8*	5’-TGACCATGAGACACTGTGGC-3’	5’-GAAGAGCACGGGTCCTTTGA-3’
*IL-10*	5’-GCGACGCTGTCATCGATTTC-3’	5’-GTAGATGCCGGGTGGTTCAA-3’
*MMP2*	5’-CCCCATGTGTCTTCCCCTTC-3’	5’-AGCTCCTGGATCCCCTTGAT-3’
*MMP9*	5’-AGGGCCCCTTTCTTATTGCC-3’	5’-CGAGTAACGCTCTGGGGATC-3’
*TIMP1*	5’-TGTGCACAGTGTTTCCCTGT-3’	5’-TAGCCCTTCTCAGAGCCCAT-3’
*GAPDH*	5’-AACTCCCATTCCTCCACCTT-3’	5’-GAGGGCCTCTCTCTTGCTCT-3’

### Statistical analyses

The differences between the obtained values (mean ± SEM, *n* = 10) were assessed by one-way analysis of variance (ANOVA) followed by Tukey–Kramer multiple comparison using Graph Pad Prism five software (GraphPad Software, Inc., La Jolla, CA, USA). The differences were considered statistically significant when *p* <0.05.

## Results

**Fig. 1:**
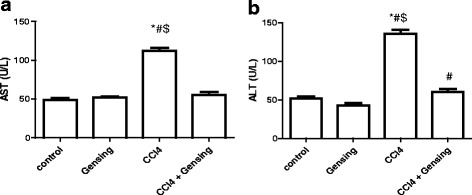
Effect of CCl4, Ginseng, and CCl_4_ + Ginseng on the serum levels of AST (a) and ALT (b). Data are presented as the mean ± SEM (*n* = 10). *, # and $ indicate a significant change from control, Ginseng and CCl_4_ + Ginseng groups, respectively (*p* <0.05 using ANOVA followed by Tukey—Kramer as a post ANOVA test)

Liver enzyme (ALT and AST) levels in sera were used as biochemical markers for early acute hepatotoxicity. The CCl_4_ group showed a significant increase in the levels of AST (112.3 ± 3.5 U/L) (Fig. 1a) and ALT (135.6 ± 5.2 U/L) (Fig. 1b) compared with the control group (48.9 ± 2.8 U/L and 52.2 ± 2.2 U/L, respectively) (*p* <0.001). However, ginseng restored liver enzyme levels to values similar to the control group.

CCl_4_ significantly increased serum lipid parameters including TG, TC and LDL levels and nonsignificantly decreased the HDL level compared with the control group. Ginseng supplementation in combination with CCl_4_ significantly decreased TC and LDL levels compared with the CCl_4_ group. No effect of ginseng alone on TG, TC, HDL, and LDL levels was observed (Table 2).

**Table 2:** Correlation between lipid profile and the studied groups

Group	TG (mg/dl)	Total Cholesterol(mg/dl)	HDL(mg/dl)	LDL(mg/dl)
Control	62.3 ± 3.99	55.6 ± 2.2	28.4 ± 1.32	44.3 ± 2.4
ginseng	58.5 ± 4.6	61.7 ± 3.5	33.2 ± 2.1	48.1 ± 2.8
CCl4	98.2 ± 3.5*#$	127.5 ± 6.5*#$	23.2 ± 1.54	93.5 ± 2.8*#$
CCl4+ ginseng	70.2 ± 3.54	70.67 ± 5.3	33.2 ± 3.32	50.2 ± 2.1

Values are the means ± SE (*n* = 10). *, # and $ indicate a significant change from control, ginseng, and CCl_4_ + ginseng groups, respectively (*p* <0.05 using ANOVA followed by Tukey—Kramer as a post ANOVA test)

### Histopathological examination of the liver

**Fig. 2:**
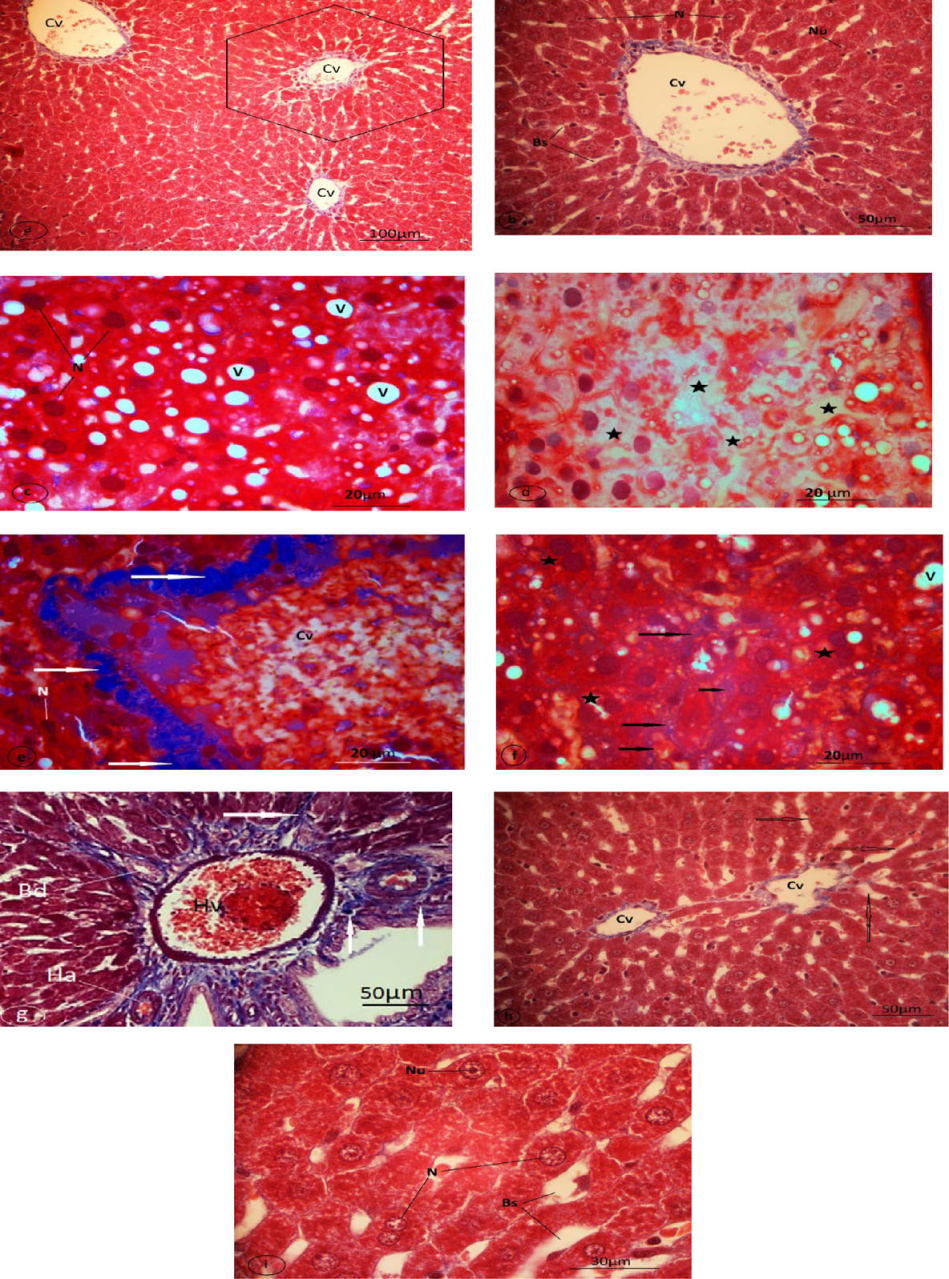
Light micrographs of liver sections of rat. Specimens were fixed in 10% neutral formalin and stained with Masson Trichrome. **a**-**b** Control rat (GI). **a** Control rat (GI) with poorly defined classical hepatic lobules and central veins (Cv) (magnification 100x). **b**: A hexagonal hepatocyte with central nucleus (N) and prominent nucleolus (Nu) arranged in strands around the central vein (Cv) and blood sinusoids (Bs) (magnification 400x). **c**-**g** Rats treated with CCl4 (GIII). **e** Hydropic degeneration and vacuolated hepatocytes. (N) Nuclei, (V) vacuoles. A marked increase in collagen fibers (arrows) around a congested central vein (Cv) and degenerated hepatocytes with nuclei (N) (magnification 1000x). **f** Fatty changes liver (stars) and collapsed sinusoids. f: Collagen fibers (arrows) around collapsed sinusoids. An increase in binucleated hepatocytes (stars) and vacuoles (V) was observed (magnification 1000x). **g** Fibrosis in the portal area (arrows). (Hv) hepatic portal vein, (Ha) hepatic artery, and (Bd) proliferated bile duct (magnification 400x). **h** Ginseng supplementation with CCl4 (GIV) reduced the accumulation of collagen fibers after CCl4 administration. (Cv) central veins, (arrows) blood sinusoids (magnification 400x). **i** Nearly normal hepatic strands and hepatocytes of GIV with nuclei (N) and nucleoli (Nu), and the reduction of perisinusoidal collagen fibers. Bs: blood sinusoids (magnification 1000x)

Histopathological abnormalities in the liver were studied at the end of the experiment using Masson’s trichrome staining. The effects of CCl_4_, ginseng, and CCl_4_ + ginseng on histopathological changes in the liver, especially in liver fibrosis, are shown in Fig. 2.

In the control group (GI), all features of the liver were observed to be normal (hepatic lobules with central veins, hepatic strands of hepatocytes, portal area, and blood sinusoids) (Fig. 2a, b). Animals in group II who received ginseng dissolved in water showed no histological evidence of pathological alterations (Fig. 2c, d,).

In the CCl_4_-treated group (GIII), the liver showed classical fibrosis around the congested central vein (Fig. 2e) and portal area, which was accompanied by a variable degree of proliferated bile duct (Fig. 2g). The normal architecture of the hepatic lobules were lost (Fig. 2f), and fibroid collapsed blood sinusoids were demonstrated. Hydropic degeneration, vacuolization (Fig. 2c), and fatty hepatocytes were observed (Fig. 2d).

In the CCl_4_ and ginseng group (GIV), the normal architecture of hepatic lobules was observed (Fig. 2h). The hepatic strands were composed of atypical polyhedral hepatocytes with a distinct nucleus and prominent nucleolus. A reduction of fatty hepatocytes, vacuoles, and collagen fibers especially around the central vein were detected (Fig. 2i).

**Fig. 3:**
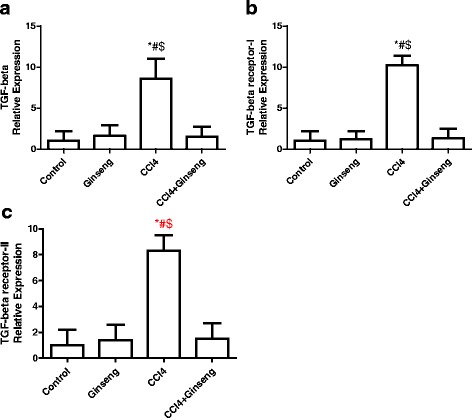
Effect of CCl_4_, ginseng, and CCl_4_ + ginseng on the expression of *TGF-β* (A), *TβF-I* (B) and *TβF-II* (C) genes in the liver. Data are the mean ± SEM (*n* = 10). *, # and $ indicate a significant change from the control, ginseng, and CCl_4_ + ginseng groups, respectively (*p* <0.05 using ANOVA followed by Tukey—Kramer as a post ANOVA test)

The effect of CCl_4_, ginseng, and CCl_4_ + ginseng on *TGF-β, TβF-I,* and *TβF-*II expression is shown in Fig. 3. The expressions of *TGF-β, TβF-I,* and *TβF-*II in the CCl_4_ group were increased significantly by 7.6, 9.2 and 7.3-fold, respectively, compared with the control group (*p* <0.001). Interestingly, the administration of CCl_4_ + ginseng resulted in a significant decrease in the expression of *TGF-β* and its receptors (*p* <0.002).

**Fig. 4:**
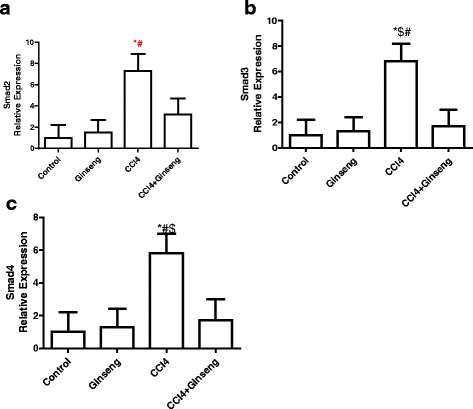
Effect of CCl_4_, ginseng, and CCl_4_ + ginseng on the expression of *Smad2* (A), *Smad3* (B) and *Smad4* (C) in the liver. Data are the mean ± SEM (*n* = 10). *, # and $ indicate a significant change from the control, ginseng, and CCl4 + ginseng, groups, respectively (*p* <0.05 using ANOVA followed by Tukey—Kramer as a post ANOVA test)

The effect of CCl_4_, ginseng, and CCl_4_ + ginseng on *Smad2, Smad3, and Smad4* expression is shown in Fig. 4. The administration of CCl_4_ for 8 weeks increased the expression of *Smad2*, *Smad3* and *Smad4* by 2.2-, 5.8- and 4.8-fold, respectively, compared with the control group (*p* <0.001). Interestingly, the administration of ginseng in combination with CCl_4_ significantly decreased the expression of *Smad2*, *Smad3* and *Smad4* by 7-, 3.5- and 5.1-fold, respectively, compared with the CCl_4_ group (*p* <0.001).

**Fig. 5:**
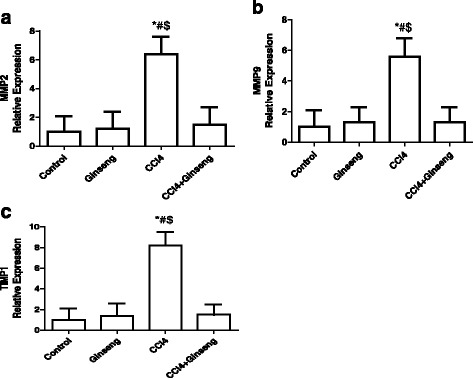
Effect of CCl_4_, ginseng, and CCl_4_ + ginseng on the expression of *MMP2* (**a**), *MMP9* (**b**) and *TIMP-1* (**c**) genes in the liver. Data are the mean ± SEM (*n* = 10). *, # and $ indicate a significant change from the control, ginseng, and CCl_4_ + ginseng groups, respectively (*p* <0.05 using ANOVA followed by Tukey—Kramer as a post ANOVA test)

The effects of CCl4, ginseng, and CCl_4_ + ginseng on MMP2, MMP9, and TIMP-1 gene expression are shown in Fig. 5. The administration of CCl_4_ for 8 weeks increased the expression of *MMP2, MMP9,* and *TIMP-1* by 5.4-, 4.6- and 7.2-fold, respectively, compared with the control group (*p* <0.001). However, the administration of CCl_4_ + ginseng significant decreased the expression of *MMP2, MMP9,* and *TIMP-1* by 5-, 4.3- and 6.7-fold, respectively, compared with the CCl_4_ group (*p* <0.0001). No significant difference was observed for gene expression in the CCl_4_ + ginseng group compared with the control group.

**Fig. 6:**
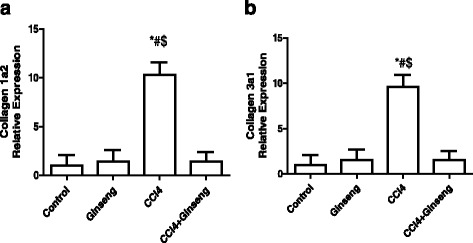
Effect of CCl_4_, ginseng, and CCl_4_ + ginseng on the expression of *Collagen 1a2* (**a**) and *Collagen 3a1* (**b**) genes in the rat liver. Data are the mean ± SEM (*n* = 10). *, # and $ indicate a significant change from the control, ginseng, and CCl_4_ + ginseng groups, respectively (*p* <0.05 using ANOVA followed by Tukey—Kramer as a post ANOVA test)

The effect of CCl_4_, ginseng, and CCl_4_ + ginseng on *Col1a2* and *Col3a1* gene expression is shown in Fig. 6a and b. The administration of CCl_4_ for 8 weeks significantly increased the expression of *Col1a2* 9.5-fold compared with the control group (*p* <0.001). The administration of CCl_4_ increased the expression of *Col3a1* 8.3-fold (*p* <0.001). However, the administration of CCl_4_ + ginseng significantly restored the alteration in these genes compared with the CCl_4_ group.

**Fig. 7:**
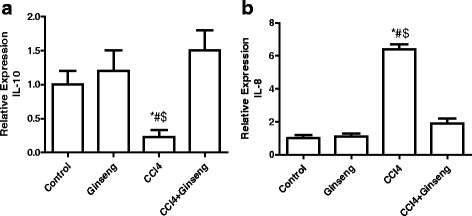
Effect of CCl_4_, ginseng, and CCl_4_ + ginseng on the expression of *IL-10* (**a**) and *IL-8* (**b**) genes in the liver. Data are the mean ± SEM (*n* = 10). *, # and $ indicate a significant change from the control, ginseng, and CCl_4_ + ginseng groups, respectively (*p* <0.05 using ANOVA followed by Tukey—Kramer as a post ANOVA test)

The effect of CCl_4_, ginseng, and CCl_4_ + ginseng on *IL-10* and *IL-8* gene expression is shown in Fig. 7a and b. The administration of CCl_4_ for 8 weeks significantly decreased the expression of *IL-10* 4-fold compared with the control group (*p* <0.05). The administration of CCl_4_ increased the expression of *IL-8* 5.4-fold (*p* <0.01). However, the administration of CCl_4_ + ginseng significantly restored the alteration in these genes compared with the CCl_4_ group. No significant difference in gene expression was observed in the CCl_4_ + ginseng group compared with the control group.

## Discussion

The liver is the first line of protection against hepatic damage induced by xenobiotics and drugs, which can cause hepatic necrosis and apoptosis [28]. Reactive oxygen species cause direct tissue damage and initiate inflammation through the activation of various cytokines [29]. Lipid peroxidation and free radicals cause the necrosis of hepatocytes, induce inflammation, and promote the progression of hepatic fibrogenesis [30].

CCl_4_ is used to induce hepatic fibrosis in experimental rat models, and several studies have focused on the prevention of CCl_4_-induced hepatotoxicity [31–33]. CCl_4_ is used to investigate the liver injury associated with oxidative stress and free radicals. Ginseng extract contains Rb1, Rb2, Rc, Rd, Re, and Rg1 that have a major role in hepato-protection by suppressing oxidative stress and lipid peroxides via inhibition of the expression and activity of cytochrome P450 in the liver [34].

The leakage of hepatocellular enzymes is used as a hepatotoxicity marker. The current study showed significant increases in the serum levels of ALT, AST, total cholesterol, triglyceride, LDL, and a significant decrease in HDL following CCl_4_ administration. However, in the CCl_4_ + ginseng group, the levels of ALT and AST were restored to their normal levels. These results indicate that ginseng protects against CCl_4_-induced hepatic damage. Similar previous studies demonstrated that ginseng extract had antioxidant activity and acted as a free radical scavenger [34, 35]. Importantly, the increased levels of triglycerides, total cholesterol, and LDL and the decreased level of HDL were restored to their normal values with CCl_4_ + ginseng.

IL-8 is as a major factor of acute inflammation [36]. High IL-8 levels in the liver are observed during acute liver injury [37, 38]. In the present study, CCl4 increased the expression of IL-8. A similar study found that serum IL-8 levels were significantly elevated in chronic liver disease patients. IL-8 serum levels were also associated with the progression of fibrosis [39]. Therefore, IL-8 overexpression might be related to tissue damage [40, 41]. In the current study, a high expression of IL-8 was observed in association with CCl4, and the administration of ginseng extract decreased IL-8 levels to normal values indicating the anti-inflammatory effects of ginseng extract.

IL-10 is a cytokine produced by cells of the immune system as well as liver cells including hepatocytes, sinusoidal cells, Kupffer cells, stellate cells, and liver-associated lymphocytes [42]. It was reported that endogenous IL-10 decreased intrahepatic inflammatory responses and fibrosis in several models of liver injury [43]. In the current study, the down-regulation of *Il-10* induced by CCl4 lead to the overexpression of collagen types I and III. Another study found that collagen types I and III were significantly decreased to normal values at 3 weeks after IL-10 treatment. Furthermore, the high expressions of MMP2 and TIMP-1 induced by CCl4 were significantly decreased after 3 weeks of IL-10 treatment, indicating that collagen types I and III might be associated with a decrease in MMP-2 and TIMP-1 levels[44]. In the current study, ginseng extract increased the expression of *IL-10,* which was associated with a decrease in MMP-2, -9 and MIT-1 and a decrease in the expression of collagen types I and III. These results indicated that exogenous IL-10 might have a therapeutic effect on advanced liver fibrosis.

Hydroxyproline, a major constituent of collagen, is a good marker for ECM accumulation [45]. The overexpression of type I, III and IV collagen are early events in the development of CCl_4_-induced hepatic fibrosis. Type I and III collagen fibers are localized around the portal area and are markedly increased around the tributaries of portal veins. In the current study, histological analysis of liver from the CCl_4_ group showed fibrosis around the central veins, portal area, and perisinusoidal space. In the current study, livers in the CCl_4_ group showed fibrosis of the central veins as indicated by collagen fibers in the portal area and highly proliferated bile duct, necrotic disorganized hepatic strands, hyalinization of cytoplasm and vacuolated hepatocytes. In addition, the results of the present study are in agreement with a study by Iredale et al., that reported mild fatty change and the vacuolization of hepatocytes after the intraperitoneal injection of rats with CCl_4_ [46].

In the present study, CCl_4_ increased the expression of collagen 1a1 and collagen 1a III. A similar study reported the increased expression of collagen types I and II after CCl_4_ administration [47]. These findings were consistent with those of Yu et al., who reported that the overexpression of type I, III and IV collagen were early events in the development of CCl_4_-induced hepatic fibrosis in rats. Type I and III collagen fibers are localized around the portal area [45] and are markedly increased around the tributaries of portal veins [47]. The overexpression of COL1A1 and CL1AIII leads to enhanced collagenous matrix deposition in liver. The CCl_4_ + ginseng group had a reduced accumulation of collagen fibers compared with the CCl_4_ group. Similarly, in the present study, the administration of ginseng extract reduced the expression of collagens type I and II.

Extracellular matrix (ECM) accumulation is a common phenomenon in liver fibrosis. MMPs and TIMPs are a class of secreted enzymes with important functions in ECM degradation [48] and MMPs are associated with liver fibrosis. MMPs and their tissue inhibitors were elevated quickly after CCl4-induced liver injury in rats, and the hepatic expression of MMP-3 was detected as early as 6 h after CCl4 administration in rats. In the current study, the administration of CCl4 caused an overexpression of *MMP-2, -9,* and *TIMP-1*. Similarly, Knittel et al., reported MMP and TIMP overexpression during liver injury and fibrosis after a single dose of CCl_4_, and that MMP-2, MMP-3, MMP-9, MMP-10, MMP-13, and MT1-MMP were all overexpressed [49]. In a rat model of hepatic fibrosis induced by bile duct ligation (BDL), MMP-2 and MMP-9 were increased suggesting that continued tissue damage and inflammation induced MMP expression [50]. Therefore, MMPs might be associated with liver fibrosis. In the present study, ginseng administration with CCl_4_ decreased the expressions of *MMP-2, -9* and *TIMP-1* to their normal levels similar to that in the control group. Similarly, Lo et al., found that ginsenoside significantly reduced the expression of MMP-2, -9 and TIMP-1 in HSC-T6 cells after induction with H_2_O_2_. They suggested that ginsenoside had an anti-fibrotic effect on HSCs by inhibiting the activation, proliferation, and expression of collagen, TGF-β1, MMP-2, and TIMP-1 [51].

The pathogenesis of fibrosis involves several mechanisms such as the inflammatory, growth factor signaling, and lipid signaling pathways. The inflammatory pathway and the growth factor signaling pathway mediated by TGF-β are the most important pathways for fibrosis [52]. Transforming growth factor-β is involved in various physiologic processes; therefore, understanding the molecular mechanisms involved in TGF-β signaling in diseases is important for the development of its therapies. The regulation of extra-cellular matrix accumulation by fibrogenic transforming growth factor (TGF)-β signals involves different mechanisms dependent upon whether there is acute or chronic liver damage. Hepatic stellate cells (HSC) are the principal effector cell type in this pathway. After acute liver injury, TGF-β enhances collagen synthesis by activating hepatic stellate cells via the Smad pathways. Activated TGF-β mediates the activation of Smad2, Smad3, and Smad4 in a fibrotic rat model [53]. In the present study, CCl_4_ increased *Smad2, Smad3, and Smad4* expression, and ginseng extract restored their expression to normal levels and decreased histological fibrosis. These results indicate that Smad2, Smad3, and Smad4 have a significant role in the progression of liver fibrosis. A similar study found that the high expression of Smad-2 and Smad-4 was associated with liver fibrosis in rats using in situ hybridization [54].

TGF-β1 is a pro-fibrogenic cytokine in hepatic fibrosis [47]. Activated TGF‐β1 signal to the cells through its trans-membrane receptors TβR‐I and TβR‐II. In the present study, CCl_4_ administration increased the expression of *TGF-β1* and its receptors *TβR-1,* and *TβR-1I*. The increased *TGF-β1*, *TβR-1,* and *TβR-II* expressions were restored to their normal values after ginseng treatment. A similar study found that CCl_4_ administration increased TGF-β1 protein levels, and its receptor levels, and this alteration in protein expression was restored by Gypenosides [55]. Another study reported that TGF-*β1* activity was increased in rats administered a single dose of 20% CCl_4_ in olive oil, and this increase was restored by a low-dose of herbal extract [56]. Another study proposed that suppressing TGF-β-induced Smad2/Smad3 phosphorylation and nuclear translocation in HSCs attenuated fibrosis [55].

When TGF‐β1 binds to it receptors (TβR‐I and TβR‐II), it catalyzes Smad2/Smad3, which enters the nucleus, binds to transcriptional factors and regulates the expression of collagen and *TIMP* genes [24, 25]. In the current study, the expressions of TGF‐β1, TβR‐I, TβR‐II, Smad2, and Smad3 were significantly increased in the CCl4 group compared with the normal group. This indicated that the TGF‐β1/Smad signaling pathway was activated during liver fibrosis. ginseng extract administration decreased *Smad2, Smad3*, *TβR‐I, TβR‐II,* and *TGF‐β1* expression to their normal values.

## Conclusions

Ginseng extract had an anti‐fibrosis effect in a CCl_4_‐induced liver fibrosis model by regulating the TGF‐β1/Smad signaling pathway via the inhibition of *Smad3, Smad2,* and *TGF‐β1* expression.

## Abbreviations

(ALT): Alanine aminotransferase
(AST): Aspartate aminotransferase
(CCl4): Carbon tetrachloride
(Col1a2): Collagen 1a2
(Col3a1): Collagen 3a1
(HDL): High-density lipoprotein
(I.P): Intraperitoneal
(ICAM)-1: Intercellular adhesion molecule
(IL-10): interleukin -10
(IL-8): interleukin-8
(LDL): Low-density lipoprotein
(MMP2): Matrix metalloproteinase 2
(MMP9): Matrix metalloproteinase 9
(NF-κB): Nuclear factor-κB
(NO): Nitric oxide
(PGE2): Prostaglandin E2
(real-time PCR): real time polymerase chain reaction
(Smad2): Mothers against decapentaplegic homolog 2
(Smad3): Mothers against decapentaplegic homolog 3
(Smad4): Mothers against decapentaplegic homolog 4
(TC): Total cholesterol
(TG): Triglycerides
(TGF-β): Transforming growth factor beta
(TIMP-1): Tissue inhibitor matrix metalloproteinase-1
(TNF)-α: Tumor necrosis factor
(TβR-1): Transforming growth factor beta receptor type I
(TβR-II): Transforming growth factor beta receptor type II
